# Genetic Analysis for Coronary Artery Disease Toward Diverse Populations

**DOI:** 10.3389/fgene.2021.766485

**Published:** 2021-11-22

**Authors:** Kazuo Miyazawa, Kaoru Ito

**Affiliations:** Laboratory for Cardiovascular Genomics and Informatics, RIKEN Center for Integrative Medical Sciences, Yokohama, Japan

**Keywords:** coronary artery disease, genome-wide association study, population diversity, biobank, precision medicine

## Abstract

Coronary artery disease is one of the leading causes of death in the world, and as such, it is one of the diseases for which genetic analyses have been actively conducted. In the early days, analyses of families with the aggregation of early-onset myocardial infarction, such as those with familial hypercholesterolemia, was the main focus, but since the practical application of genome-wide association study, the analysis of coronary artery disease as a common disease has progressed, and many disease-susceptibility loci have been identified. In addition, with the advancement of technologies, it has become possible to identify relatively rare genetic variants in a population-based analysis. These advances have not only revealed the detailed disease mechanisms but have also enabled the quantification of individual genetic risk and the development of new therapeutic agents. In this paper, some of those items, which are important to know in the current genetic analyses for coronary artery disease, are discussed.

## Introduction

Coronary artery disease (CAD) causes myocardial ischemia due to narrowing or blockage of the coronary arteries that feed the heart based on atherosclerotic predisposition, leading to myocardial infarction (MI). The disease can also lead to the development of arrhythmia, heart failure, and death. Epidemiologically, CAD is one of the leading causes of death in the world, affecting approximately 1.72% of the world’s population, with nine million deaths reported in 2017 (Khan et al., 2020). Such high morbidity and mortality rates have a significant impact in terms of medical economic and social burden, which urgently needs to be addressed. Therefore, genetic studies for CAD and attempts to apply the results to clinical practice are being spearheaded around the world.

### Advances in the Genetic Analyses for Coronary Artery Disease

The history of genetic research on CAD began with the analysis of families that suffered early-onset MI. In the familial analysis, the method called linkage analysis was mainly used, which assumes that the disease is caused by a single, high-penetrance genetic mutation and is transmitted from generation to generation according to Mendelian laws. Diseases with this characteristic are also called monogenic disorders, but this form of inheritance has not been observed in many patients with CAD and only explains some of the genetic factors. However, family-based analysis of the disease was a very effective tool not only for elucidating the pathogenesis of the disease but also for drug discovery, because it allowed us to observe a clear phenotype leading to the development of the disease and to identify the causative genes. One of the earliest reports of such family-based analysis was of a patient with familial hypercholesterolemia (FH) who had early-onset MI as well as abnormally high cholesterol levels and xanthoma. The discovery of such families with a *LDLR* gene mutation led to the subsequent identification of genes associated with FH (Vrablik et al., 2020), including *APOB*, *PCSK9*, *LDLRAP1*, *ABCG5*, and *ABCG8*. In addition, *LRP6* gene mutations associated with LDL cholesterol, triglycerides, hypertension, diabetes mellitus, and osteoporosis (Mani et al., 2007) and *DYRK1B* gene mutations associated with obesity, severe hypertension, and diabetes mellitus (Keramati et al., 2014) have been reported as familial-onset MI genes that are different from FH and cause MI at a young age.

**FIGURE 1:**
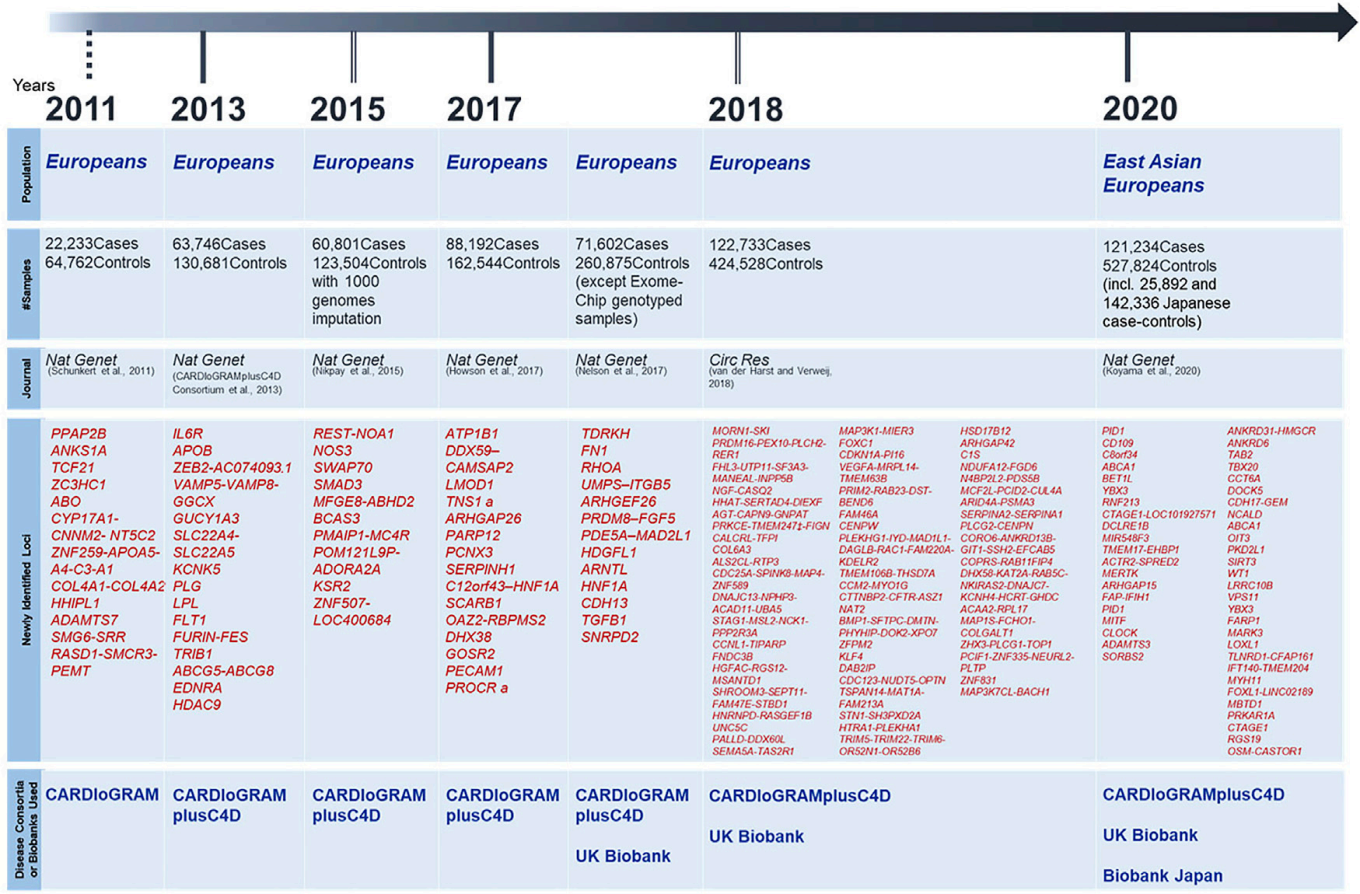
Landmark papers of CAD-GWAS and Newly Identified Disease Susceptibility Loci Since 2010.

On the other hand, since the majority of patients with CAD do not show clear Mendelian heredity, it was necessary to conduct a genetic analysis of these patients using another method. Although the linkage analysis did not work well for such samples, Tanaka, Ozaki and their colleagues were the first in the world to conduct a genome-wide association study (GWAS) using the information on approximately 90,000 single nucleotide polymorphisms (SNPs), followed by identifying a disease-susceptibility locus (Ozaki et al., 2002). The SNP identified in the study was on the lymphotoxin-alpha (*LTA*) gene located in the HLA region of chromosome 6q21, which encodes a pro-inflammatory cytokine. Subsequently, several SNPs related to inflammation were identified, strongly suggesting that the inflammatory cascade was very important in the pathogenesis of CAD. In the early days, GWAS were performed on a scale of a few hundred individuals or less, but with the emergence of disease consortia and huge biobanks, the sample size has rapidly increased. Looking at landmark papers from the last decade, as shown in Figure 1, the study conducted by CARDIoGRAM consortium in 2011 comprised approximately 90,000 individuals (Schunkert et al., 2011), the study conducted by CARDIoGRAMplusC4D consortium in 2013 comprised approximately 190,000 individuals (Consortium et al., 2013), and Nikpay et al. performed a GWAS in 2015 using the whole genome sequencing (WGS) data from the 1,000 Genomes Project as a reference panel for imputation to test about 10 million variants (Nikpay et al., 2015). In 2017, Howson et al. (2017) encompassed about 250,000 samples while Nelson et al.’s study included approximately 320,000 samples (excluding exome-chip analysis samples) (Nelson et al., 2017), and van et al.’s study in 2018 increased the sample size to about 400,000 (van der Harst and Verweij, 2018). However, most of the samples in those studies so far have been collected from European populations. The ethnic specificity of genetic architecture has already been discussed, and considering the clinical application of genetic analysis results, each ethnic group needs to have its own evidence of genetic research to rely on. In 2017, Lu et al. (2017) analyzed 47,532 East Asians for lipid levels, which are heritable risk factors for CAD, using exome arrays and identified three chip-wide disease susceptibility loci. They further compared these results with a GWAS of 28,899 Chinese subjects with CAD and a GWAS of approximately 190,000 subjects in the CARDIoGRAMplusC4D consortium, and found rs7901016, a non-coding variant near *MCU* gene, lower LDL cholesterol level and reduce the risk of CAD. In 2020, a Japanese group conducted a GWAS using approximately 180,000 Japanese and identified eight disease-susceptibility loci that had not been identified in larger European studies, which demonstrated the importance of genetic analysis in non-European populations. In addition, they conducted a meta-analysis with European GWAS with a total of 650,000 subjects (Koyama et al., 2020), which is the largest scale analysis in the world so far, and identified 35 novel disease-susceptibility loci. As such, like the situation of GWAS for other diseases, genetic studies for CAD have shown a rapid increase in sample size and a concomitant rapid increase in the number of disease-susceptibility loci.

### Chasing Rare Variants in the Population-Level Analysis

The existence of inexpensive genotyping arrays for GWAS of CAD makes it possible to increase the sample size, and so far such attempts have been very successful. However, genotyping arrays have a limited number of probes and the genomic information that can be obtained at a time is limited. On the other hand, family-based analysis identifies rare genetic variants with strong effects, which are difficult to capture with genotyping arrays targeting common variants. Genetic variants detected in family-based analysis are very rare, and sequencing was necessary to detect such rare variants at the population level. However, the identification of rare variants continued to be difficult due to the high cost and difficulty in increasing the number of samples compared to genotyping arrays. Thus, there is a large gap between the search for common and rare variants, and the identification of rare variants at the population level has been considered to be difficult.

One of the solutions is to perform WGS on all samples, although the cost is much higher. Currently, the only large-scale WGS project is the Million Veterans Program’s 100,000 (as of February 2021: https://www.darkdaily.com/vas-million-veterans-program-research-study-receives-its-100000th-human-genome-sequence/), and the TOPMed project led by the U.S. National Heart, Lung, and Blood Institute (160,000 people as of Freeze 8, 2021: https://www.nhlbiwgs.org/topmed-whole-genome-sequencing-methods-freeze-8), etc.

On the other hand, there is a method called imputation that uses haplotype information obtained from WGS as a means of obtaining genomic information that is not as sparse in terms of variant information as genotyping arrays, although it does not have the near-complete coverage of WGS. In this method, haplotype information from WGS is used as a reference panel, and variant information typed by genotyping arrays is used as a scaffold to predict untyped regions. For a long time, the WGS information of 2,504 individuals (Phase 3) of the 1,000 Genomes Project was used as the golden standard as the reference panel for imputation. However, since the panel covered Europeans, Americans, Africans, and East Asians equally, it did not have enough information on haplotypes specific to each ethnic group (just several hundred individuals for each group) to impute variants with low allele frequencies. In Europe, the Haplotype Reference Consortium (HRC) was launched, whose imputation reference panel using the WGS information of about 65,000 people has been established since the initial goal. Currently, more precise imputation is possible by incorporating the WGS data of the TOPMed project mentioned above.

However, because of the ethnic specificity of haplotype information, there is little advantage for other ethnic groups to use the HRC, which is mainly used by Europeans. In a recent landmark paper on GWAS for CAD (Koyama et al., 2020), they not only used the WGS data of 4,930 Japanese but also created a reference panel containing disease-specific haplotype information for 1,782 patients with CAD for imputation. Due to the advantageous conditions such as the unity of the Japanese population and disease specificity, their imputation was able to include variants with allele frequencies of about 0.02% in the analysis with less than one-tenth of the WGS data samples of HRC. As a result, they identified a missense mutation in the *RNF213* gene, which has been reported as a causative gene of Moyamoya disease, for the first time in the population analysis of CAD. In addition, they identified a group of genetic variants that cause FH, including protein-coding variants in the *LDLR*, *PCSK9*, and *APOB* genes. The magnitude of the effect on the onset of the disease and the effect on the age of disease onset were also shown at the population level. This identification and characterization of variants previously identified in family-based analyses at the population-level using genotyping arrays prove that population-level analysis is now able to approach rare variants that were previously within the scope of genealogical analysis. Thus, imputation is a cost-effective method that is still frequently used to reuse genotyped array samples but will be replaced by WGS in the not-too-distant future.

### From Genetic Variants to Functional Analysis

In contrast to monogenic disorders, disease-associated SNPs detected by GWAS are often located in non-protein-coding regions, and the presence of linkage disequilibrium (LD) blocks means that lead SNPs are not always the cause of the disease. It is not easy to infer the exact mechanism leading to the disease phenotype from such SNPs. For example, the chromosome 9p21 region, which is a locus particularly strongly associated with CAD, does not contain protein-coding genes, making it difficult to elucidate the mechanism leading to the disease development. Although candidate genes such as *ANRIL* (Lo Sardo et al., 2018), a long non-coding RNA, and *CDKN2A* and *CDKN2B* (Kojima et al., 2014), which are located in the vicinity of *ANRIL*, were identified in several wet experiments, no conclusion has been reached as to which gene or mechanism is responsible. Chromosome 6p24 is also a disease susceptibility locus strongly associated with CAD, carotid artery dissection, and hypertension, and recent omics analyses have reported several disease pathogenic mechanisms. 6p24 has two candidate genes, phosphatase and action regulator 1 gene (*PHACTR1*) and endothelin-1 gene (*EDN1*). Although rs9349379, the lead SNP of this region, is located in the intron region of *PHACTR1*, the effect of PHACTR1 protein on the pathogenesis of CAD was unclear since the past molecular biological studies. On the other hand, EDN1 protein was well known as a protein involved in atherosclerosis. Expression quantitative trait loci analysis (eQTL analysis) using vascular tissue showed a strong correlation between rs9349379 and *PHACTR1* gene expression (Beaudoin et al., 2015). On the other hand, when rs9349379 was introduced into induced Pluripotent Stem Cells (iPSC)-derived vascular-like cells using genome editing technology, a strong correlation was observed between rs9349379 and the expression of the *EDN1* gene, suggesting that *EDN1* is the causative gene (Gupta et al., 2017). Thus, conflicting results were obtained depending on the experimental system, and it is necessary to accumulate more evidence to determine which of these genes is involved in the disease development, or both. Thus, the number of causative genes and their independence in a single disease-susceptible locus are often unknown, and even if immortalized human cultured cells or iPSC are used as a human model, and their detection is difficult due to their small effect. In addition, repeating such experiments for each individual cell would require a large amount of monetary and time resources. Therefore, there is an urgent need to establish a fast and multiplex experimental system that can reproduce the human environment and detect the effects of many genetic variants at once.

### Realization of Precision Medicine From Genomic Analysis

Genetic risk identified by genomic analysis is a risk factor to which we are constantly exposed from birth to death, unlike lifestyle risk that becomes apparent after we reach adulthood. If this information can be used to predict the disease onset, assess the disease severity, and intervene in treatment, it will be a great help in achieving precision medicine. Various studies/researches for this purpose are currently being actively conducted around the world. Unlike monogenic disorders, in which a single genetic variant can be a definitive marker for the diagnosis, CAD is mainly developed by the accumulation of many common variants with weak effects and high frequency. To predict the genetic risk of such a disease, it was necessary to integrate information from multiple genetic markers, and the genetic risk score (GRS) was devised for this purpose. The GRS is calculated as the sum of (the number of an alternative allele multiplied by the amount of effect of a variant on the disease onset as estimated by GWAS), using the lead variants that meet the genome-wide significance level in GWAS as markers. Recently, given the fact that including more variants improves the performance (to some extent), a polygenic risk score (PRS) has been developed, in which thousands to tens of millions of variants are included. Since GRS is a concept that includes PRS, PRS will also be referred to simply as GRS in this paper.

A landmark paper demonstrating the usefulness of CAD-GRS was reported in 2016, which examined the contrast with lifestyle risk (Khera et al., 2016). This paper showed that the hazard ratio of developing coronary events in the top 20% of the GRS group was similar to that in the group of patients with unfavorable lifestyle habits. The paper also showed that even with a high GRS, a healthy lifestyle was able to offset the genetic risk (45% relative risk reduction), indicating not only the usefulness of the GRS but also that aggressive intervention was effective in reducing coronary events in patients with a high GRS. The next important paper demonstrating the usefulness of GRS was published in 2018 (Inouye et al., 2018). The authors proposed a new framework to improve the performance of GRS, where they showed that the derived GRS was better than existing clinical risk factors in predicting the CAD onset and that the integration of GRS and clinical risk scores further improved the performance. Finally, the therapeutic utility of the GRS for CAD was demonstrated in a paper (Kullo et al., 2016), where the authors followed LDL cholesterol levels, considered one of the strongest risk factors for the disease, in 203 patients at intermediate risk for CAD who were not taking lipid-lowering drugs such as statins, in two groups: one with the clinical risk score and the other with both the clinical risk score and the GRS. After 6 months, LDL cholesterol levels were significantly lower in the group that received both scores, and there was a particularly strong reduction in LDL cholesterol levels in the group with the high GRS. Although this is not a study of a protocol that mandates therapeutic intervention according to the GRS, it is an example of how the presentation of this information to patients and their physicians could have a strong influence on subsequent treatment decisions.

Although the CAD-GRS is expected to be helpful in clinical practice, there are concerns about its applications. The study design of the GWAS from which the GRS is derived influences the characteristics of the GRS. This could be the definition of cases and controls, or it could be the issue of population specificity. Regarding the former, a position paper published in Nature 2021 calls for transparency and disclosure of information in the derivation, performance evaluation, and validation of the GRS (Wand et al., 2021). In fact, regarding the additive effect of CAD-GRS, Mosley et al. published a paper demonstrating no improvement in the performance by adding CAD-GRS to a clinical risk score (Mosley et al., 2020), contrary to the previous report (Inouye et al., 2018). Therefore, the use of GRS should be carefully evaluated to ensure that an appropriate GRS is being used and that the evaluation is fair. Concerning the latter issue of ethnic specificity, the current situation that European studies dominate the majority of academic journals gives us few choices other than using European GWAS for the GRS development. Thus, the importance of genomic analysis for other populations is now being emphasized. On the other hand, a Japanese group reported that utilizing a trans-ancestry meta-analysis with European GWAS as a GRS-derivation GWAS improved the performance of CAD-GRS, which implied that non-European populations could make use of the abundant European GWAS results for their GRS development utilizing trans-ancestry meta-analysis. As such, GRS is awaiting not only the improvement of its performance but also the emergence of new methods to deal with various populations.

### From Genome Analysis to Therapy

Genomic analysis can identify disease-related genes, and the results are expected to lead to therapeutic applications; causative genes are inferred from disease-susceptibility loci in GWAS, and drug repositioning has been performed using gene-drug interaction databases (Okada et al., 2014). However, genes derived from single-gene diseases, which provide clear information on the pathogenesis of the disease and its accompanying symptoms, are more likely to reach the clinical stage because their efficacy and adverse reactions are easier to predict when targeted for therapy than genes identified by GWAS. A recent representative example is a human monoclonal antibody against the *PCSK9* gene identified in the genomic analysis of FH, which shows a strong lipid-lowering effect. In addition, based on a report of a family lineage with mutations in the *ANGPTL3* gene (Musunuru et al., 2010) that showed low LDL cholesterol, low HDL cholesterol, and low triglycerides, antibodies (Dewey et al., 2017) or antisense oligonucleotides (Graham et al., 2017) targeting the gene are being developed (the similar trials are being performed for the *ANGPTL4* gene). In addition, although not limited to CAD, CRISPR/Cas9, which enables easier gene modification, or methods that avoid DNA double-strand break and reduce off-target effects, and improve safety, have recently emerged.

## Discussion

Genomic research on CAD is steadily expanding the horizon, with the help of the emergence of huge biobanks as well as the activities of powerful disease consortia. Furthermore, CAD-PRS is beginning to pave the way for clinical applications. Because of the various risk factors like so-called lifestyle-related diseases such as hypertension, diabetes, and dyslipidemia, and because of the several sub-forms such as stable angina, acute coronary syndrome, and vasospastic angina, the mechanisms of the disease development are expected to be very diverse, and there are still many issues to be addressed for further research. However, as mentioned, populations other than Europeans are underrepresented, and it will be necessary to pay more attention to population diversity as well as to the scale and technology of the research.

In the near future, WGS will play a leading role in the genetic analysis of CAD, and as the corresponding information in other omics layers becomes more complete, CAD genome research will continue to develop. It is hoped that the results will lead to the clinical implementation of precision medicine for CAD.
